# Medical Opinion and Movement

**Published:** 1911-04-01

**Authors:** 


### Medical Opinion and Movement.

### Magnesium Sulphate for Erysipelas.

Dr. Tucker, of Philadelphia, first tried compresses of magnesium sulphate with a view to relieve the pain in certain complications of venereal diseases, such as gonorrhœal arthritis, epididymitis, etc. He was surprised to find that they not only relieved the pain, but also exercised a curative effect upon the inflammatory processes. Since then he has applied these compresses in cases of erysipelas with equally good results. In nineteen cases of this disease, complicated with alcoholism, acute nephritis, pneumonia, etc., he has had three deaths, and in thirty-five cases without any such complications the condition has completely cleared up in the course of two to seven days, without recourse to any internal medication. Dr. N. H. Choksy, of Bombay, reports equally successful results in thirty cases of erysipelas. Generally improvement is felt in the course of the first few hours, and in one to three days the pain and swelling are considerably diminished and the temperature falls to normal. The affected part is covered with about a dozen layers of gauze dipped into a saturated solution of magnesium sulphate, previously filtered through muslin. Over this is placed a piece of protective silk or guttapercha tissue, and the gauze is wetted again every two hours or even more frequently if necessary. The compresses should be changed and the part examined every twelve hours.

### Anæmia and Respiratory Trouble.

In a contribution to the *Wiener Medizinische Wochenschrift*, Dr. L. Hofbauer discusses the cause of respiratory disturbances in conditions of anæmia. His attention was drawn to the subject by a patient who, together with severe anæmia, showed signs of considerable respiratory trouble, although there was no evidence either during life or at the autopsy, apart from the anæmia, to account for this condition. By experiments on animals Dr. Hofbauer is led to the conclusion that this sort of respiratory disturbance is not due to the anæmia itself, as is generally supposed, but to the attendant low blood-pressure. If an acute anæmia is produced in an animal by a sudden severe loss of blood, a change in the respiration takes place at once, similar to that noted in the anæmic patient. If, however, the loss of blood takes place slowly, this respiratory disturbance does not show itself. Dr. Hofbauer further confirmed this hypothesis, that the respiratory disturbance was due to a fall in the blood pressure. He found that if an animal was bled severely by opening the aorta below the diaphragm the respiratory disturbance at once appeared, but as soon as the fall of blood pressure was stopped by ligature of the aorta, respiration returned to the normal type. In rabbits a similar respiratory disturbance can be produced without loss of blood by excitation of the depressor nerve and consequent fall of blood pressure. As soon as the excitation ceases, the blood pressure rises and the respiration returns to normal.

### Convulsions after Orthopædic Operations.

About a year ago Dr. Schanz drew attention to the fact that convulsive fits are liable to follow orthopædic operations, especially in young children which may prove fatal. The hypothesis offered to explain these convulsions was that they were due to fatty emboli caused by the liberation of fat from the bone marrow into the general circulation. On this hypothesis Dr. Schanz proposed to treat the condition by intravenous and subcutaneous injections of saline solution. In a recent communication to the *Deutsche Medizinische Wochenschrift*, Dr. Codivilla puts forward a different explanation of the phenomenon. He has observed nine cases of the condition, and he attributes the convulsions to the excessive tension to which the soft parts of the extremities, including the nerve-trunks, are subjected in consequence of the traction often necessary to correct the deformity. Reflexly, this tension of the nerve-trunks sets up a condition of excitation in the central nervous system which finds expression in the convulsions. This hypothesis receives confirmation from the experimental research of Dr. Neri. He has been able to produce sudden attacks of convulsions in animals by exercising strong traction on the soft parts of one or both of the lower extremities. In cases in which traction is applied to both limbs the convulsive phenomena are much more frequent and more severe. According to Dr. Codivilla these convulsions are more likely to occur in patients with some neurotic taint, or predisposed to epileptic attacks. From a therapeutic point of view Dr. Schanz's method of treatment with saline injections would appear irrational according to this latest causal theory, although Dr. Codivilla suggests that an increase in the volume of blood might assist the nerve-centres by increasing the nutrition and organic changes of the tissues, but he thinks the most important principle of treatment consists in relieving at once the tension and traction on the tissues. He suggests further that in cases in which there is some neurotic taint or liability to epilepsy, any manipulation should be preceded by a course of bromide, and that any extension or traction of the soft parts that may be necessary should be carried out gradually.

### Pulmonary Tuberculosis.

The method of treatment of pulmonary tuberculosis, introduced by Forlanini, by producing an artificial pneumo-thorax by means of intrapleural injections of nitrogen, is already well known and has been commented upon in these columns. Drs. Lyonnet and Piéry, in an interesting contribution to the *Lyon Médicale*, discuss the measures adopted by them in order to prevent certain accidents which may otherwise happen and have brought the method somewhat into discredit. The most important are the reflexes of pleural origin, which may in themselves prove fatal. In order to prevent this they inject 0.01 gramme of morphia subcutaneously before the operation. In consequence, in the course of more than sixty injections of nitrogen, they have not observed any signs of severe pain, nor general malaise, nor tendency to syncope, nor change in the pulse, nor dyspnœa, in spite of the fact that they inject 600 to 800 cubic centimetres of nitrogen, which is more than double the volume recommended by Forlanini. The next complication in order of severity is gaseous emboli by penetration of the nitrogen into the capillaries. The authors think that the importance of this complication has been exaggerated, but, according to them, it can be completely obviated by avoiding pleural adhesions and making the injections where the adhesions are least likely to be present—namely, at the anterior border or at the lower border and bases of the lungs. Further, it should be ascertained that the respiratory murmur is a little exaggerated at the point chosen for injection, as that indicates the presence of a compensating emphysema and therefore negatives any pleural adhesions. Finally, in order to further ensure against penetration of gas into the blood vessels, they use an apparatus by which aspiration can be performed before injection, and injection is only carried out if previous aspiration does not show the presence of blood. To prevent compression they do not exceed a pressure of 5 to 6 centimetres of water measured by a manometer.

### The Treatment of Residual Urine.

In an interesting note contributed by Mr. Miller to the *Edinburgh Medical Journal* he recalls a suggestion which he first made more than ten years ago for the benefit of those with commencing symptoms of enlarging prostate. By perseverance he thinks the bladder can be trained in the art of expelling residual urine, whereby the catheter life may be avoided or postponed for a long time. After passing all the urine he feels that he conveniently can, the patient is instructed to wait two or three minutes and then to try to pass some more. If as much as an ounce is expelled, this is proof that there is some residual urine in the bladder. At first the second effort should be made at every act of micturition, except during the course of the night. After a while it will be found that twice a day will suffice—first thing in the morning and last thing at night. In some cases, after a while the residual urine ceases to exist, so effectively does the bladder learn the art of emptying itself; in others it is reduced to a drachm or two; in others the effect is limited to prevention of its increase. But always greater comfort is obtained, and the unpleasant night rising is diminished, if not abolished. Since first publishing the idea the author has received many testimonials from medical men of its usefulness, and has also proved on his own person the possibility of avoiding a threatened catheter-existence.

### Inspection of the Stomach.

To the members of the laryngological section of the Royal Society of Medicine was demonstrated lately by Drs. Hill and Herschell their method of œsophago-gastroscopy by a combination of the direct and indirect methods; a full account is given in the *Journal of Laryngology*. The direct stomach-tube, provided with an inflating apparatus, is passed under cocaine into the stomach; the cardiac end of the stomach can then be examined by the direct method. Then a periscope is passed into the tube, and the pylorus itself can thus be viewed. The essence of the plan is that the gastroscope is not passed into the stomach blindly; the stomach contents can be aspirated and the viscus washed out under inspection. The authors are at present learning the normal appearances of the interior of the stomach; they have not yet begun to use it for diagnosis, as far as the pyloric region is concerned. In acute ulcer and in hæmorrhage they hesitate to recommend it; but it is thought that in the differentiation of chronic ulceration from malignant disease the method will prove of great assistance, and, in addition, be absolutely safe. The stomach is not washed out beforehand, but the patient is examined fasting. Possibly when cases of pyloric obstruction come to be dealt with it may prove necessary to revise this portion of the technique.

### Iodides in High Blood Pressure.

In the pages of the *Edinburgh Medical Journal* is printed a most useful and practical research by Dr. Matthew into the effect of iodides in reducing high blood-pressure and into the comparative values of various organic iodine compounds which are extensively advertised as proprietary products by various enterprising Continental and American firms of manufacturing druggists. The author begins by recounting the unsatisfactory results following the use of nitrites, benzoates, hippurates, diuretins, thyroid extract, mercurials, Truneck's serum and other reputed remedies for hypertension: all these he tried without permanent benefit, except that mercurials sometimes did good by removing the source of an intestinal toxæmia. The rationale of the undoubted effect of iodides in reducing blood pressure has been much debated. Romberg, and afterwards Muller, thought it to be solely due to diminished viscosity of the blood. Huchard ascribed it to vaso-dilatation; Janeway, to an action on the vessel walls. Rolleston holds that the effect is indirect, and due to a stimulation of the internal secretion of the thyroid gland. The author concludes that iodides have a specific action in cases of high blood-pressure before arterio-sclerosis has set in, but that they have no effect when once the latter condition is well established. The vaso-dilatation is, he thinks, exactly similar in kind to that of nitrites, but slower in onset and much more prolonged. This action of the iodides is useful in the earlier stages of chronic nephritis, for so-called idiopathic high blood-pressure, and for aneurism.

Dr. Matthew then proceeds to the really more important, because more novel, part of his paper, and examines critically the claims made on behalf of the organic iodides, of which he selects for investigation five—namely, tiodine, iodoglidine, sustenin, iodipin, and sajodin. The method of judging them was to administer such a dose as would contain 1.5 grain of iodine to patients with marked hypertension, and then to test the urine for iodine. From the table given it appears that the first of these preparations never caused the appearance of any iodine in the urine at all, though the experiment was several times repeated. The second, third, and fourth cause iodine to appear in the urine just as soon and to remain just as long as when the inorganic salts are given. Of the fifth it does seem to be true that elimination is delayed, and therefore that the results of administration may be rather more lasting. When doses were given t.i.d. in the usual way, the same results were obtained; sajodin established a slight but definite superiority over the others, but the inorganic salts appeared to act just as well. Clinically, sodium and potassium iodides were far superior to any of the organic salts; and the latter are of course many times as expensive and require to be given in much larger doses. As for iodism, the author holds this to be a matter of idiosyncrasy; and he attributes any smaller incidence of iodism among patients taking organic iodides solely to the fact that as a rule the doses given contain less iodine than the ordinary salts. For patients whose stomachs will not tolerate the latter, he finds sajodin sometimes useful, as it is tasteless. For hypertension his recommendation is ten grain doses of potassium iodide, rapidly increased if necessary.

### Shock and Anæsthesia.

The old question whether surgical shock is the greater when the operation is performed under light or under deep anæsthesia is reconsidered in the *Therapeutic Gazette* by Drs. Lull and Turner. These observers made extensive blood pressure and pulse-rate observations upon patients before, during, and after operation. They find that anæsthesia by ether causes in many cases a marked alteration in the ratio between the blood pressure and the pulse-rate. This is augmented by anything that produces shock to the nervous system during operation, notably incision of the skin or peritoneum, or drawing on the pedicle of an organ. If at the time of this shock anæsthesia be light, the shock will not be as marked as if the patient be under deep anæsthesia. This conclusion, which is supported in this country by the teaching of Dr. Hewitt, is contrary to what many surgeons believe. It is often taught that when the peritoneum is to be incised, or any similar shock-producing process carried on, the anæsthetic should be pushed in order that shock shall not be so severely experienced. The authors say that this is erroneous, as non-lethal doses of anæsthetics have practically no influence on the spinal cord. They advise rather that the anæsthetic be withdrawn at these critical moments of an operation.

### Autoserotherapy by Intravenous Injection.

Autoserotherapy has been tried in France and elsewhere lately for pleural effusion, hydrocele, and ascites. Up to now the fluid injected has been small in amount, a few c.c. merely, and has been introduced by the subcutaneous route into tissues in close proximity to the affected parts. Sicard and Galup at a recent meeting of the Société Médicale des Hôpitaux described another method of arriving at the same result. Instead of the subcutaneous they have made use of the intravenous route, and instead of injecting a few c.c. of the serous fluid, have made use of massive injections varying from 300 to 500 c.c. at a time. So far they have only tried the new method on two cases of ascites resulting from simple atrophic cirrhosis. One case has recovered completely and the other has improved but is still under treatment. The curious point in their report is that five or six injections were needed before any marked improvement was noticed. These were repeated every ten to fourteen days into a vein of the arm. The cured case was under treatment for four months in all, and both patients had been subjected previously to repeated tappings, subcutaneous injections, diuretics, etc., without avail. The new method is said to be painless, safe, and free from complications. The authors insist, however, that the fluid should be tested by injection into the guinea-pig so as to ascertain that it is free from bacilli before being used as an intravenous injection into the patient himself.

### The Action of Urinary Antiseptics.

Dr. Anson Jordan contributes an interesting paper on the action of urinary antiseptics to the *Bio-Chemical Journal*. The drugs chosen for experiment were urotropine, sandal-wood oil, its derivative santalol-salicylate, and salicylic acid. The micro-organisms selected for the tests were putrefactive organisms, staphylococcus pyogenes aureus, and bacillus coli communis. The urine used in all tests was a twenty-four hours specimen collected from the same individual under similar conditions of diet and exercise. The acidity and alkalinity of the urine was varied by taking respectively acid sodium phosphate or potassium citrate by the mouth. It was found that the antiseptic power of urotropine in alkaline or neutral urine is almost nil, but that the power rapidly rises as the acidity increased. Outside the body the antiseptic power of the drug is greatly increased by the addition of acid, but destroyed by that of alkali. In neutral solution urotropine disintegrates, yielding a small amount of formaldehyde (from 0.05 to 0.1 per cent.). No dissociation occurs in alkaline solution, but some 10 per cent. is converted into formaldehyde in acid solution. Sandal-wood oil was found to have a very feeble action against putrefaction, and the bacillus coli grew readily in urine containing the drug. It has a powerful antiseptic effect, however, as regards the staphylococcus, and in alkaline urine is more efficient against this organism than is urotropine. If this selective action on the staphylococcus applies to cocci generally, it would account for the results obtained by sandal-wood oil in gonorrhœa. Santalol-salicylate has a feebler action than the oil, but can be tolerated in much larger quantities. Salicylic acid gives no appreciable increase in acidity in the twenty-four hours.

